# The Intersection of Psoriasis and Neoplasia: Risk Factors, Therapeutic Approaches, and Management Strategies

**DOI:** 10.3390/cancers16244224

**Published:** 2024-12-18

**Authors:** Larisa-Alexandra Mateescu, Alexandra-Petruța Savu, Costina-Cristiana Mutu, Cezara-Diana Vaida, Elena-Daniela Șerban, Ștefana Bucur, Elena Poenaru, Alin-Codruț Nicolescu, Maria-Magdalena Constantin

**Affiliations:** 1 2nd Department of Dermatology, Colentina Clinical Hospital, 020125 Bucharest, Romania; cristiana.terziu@gmail.com (C.-C.M.); cezara-diana.vaida@rez.umfcd.ro (C.-D.V.); elena-daniela.serban@drd.umfcd.ro (E.-D.Ș.); stefana.bucur@umfcd.ro (Ș.B.); maria.constantin@umfcd.ro (M.-M.C.); 2 Faculty of Medicine, “Carol Davila” University of Medicine and Pharmacy, 050474 Bucharest, Romania; elena.poenaru@gmail.com (E.P.); nicolescualin66@yahoo.com (A.-C.N.); 3 EgoClinic, District 1, 010235 Bucharest, Romania

**Keywords:** psoriasis, neoplasia, cancer, treatment

## Abstract

The association between psoriasis and increased cancer risk is gaining recognition as studies reveal shared inflammatory and immune pathways. This review examines the relationship between psoriasis and neoplasia, focusing on cancer risk factors in psoriasis patients, the biological pathways underlying this connection, and the impact of various psoriasis treatments on cancer development. Psoriasis patients have a heightened incidence of certain cancers, such as lymphomas, skin cancers, and urological malignancies, potentially linked to immune dysregulation and chronic inflammation. Immunomodulatory treatments for psoriasis, including conventional systemic therapies and biologics, present varied cancer risks, with others, such as phototherapy, associated with an elevated risk of skin cancers. For oncologic patients with psoriasis, management necessitates a tailored approach, balancing effective psoriasis control with minimizing cancer progression risks. The emergence of IL-17 inhibitors, IL-23 inhibitors, and small-molecule therapies offers promising therapeutic alternatives with favorable safety profiles for these patients. This review underscores the need for interdisciplinary collaboration to optimize care for patients managing both psoriasis and malignancy.

## Simple Summary

Psoriasis, a chronic inflammatory skin condition, is increasingly recognized for its complex interactions with various systemic diseases, including neoplasia. This article reviews the relationship between psoriasis and neoplasia, focusing on the risk factors for cancer in psoriasis patients, the implications of psoriasis treatments on cancer risk, and strategies for managing patients with both conditions.

## 1. Introduction

Psoriasis is a chronic, inflammatory, immune-mediated condition that affects 2–3% of the total world population [1]. The prevalence varies according to age, gender, ethnicity, and geography, ranging from 0,91% (United States) to 8,5% (Norway) in the adult population and from o% (Taiwan) to 2,1% (Italy) in children [2].

Psoriasis is now recognized not just as a skin condition but as a complex disease with systemic involvement, particularly in severe forms that are associated with a higher incidence of comorbid conditions such as cardiovascular diseases, diabetes, metabolic syndrome, Crohn’s disease, ophthalmological changes, psoriatic arthritis, and depression [3,4,5].

A less clear connection is the risk of developing cancers, especially site-specific cancers, in individuals with psoriasis. Various mechanisms contribute to the pathogenesis of psoriasis, leading to persistent local and systemic inflammation that has been previously linked to an increased risk of developing neoplasia [6]. In addition, severe cases of psoriasis are managed with immunosuppressive medications, which can also elevate the risk of neoplasia.

This article examines the connection between psoriasis and neoplasia, emphasizing the cancer risk factors in psoriasis patients, the impact of psoriasis treatments on cancer development, and approaches for managing patients with both conditions.

## 2. Psoriasis and Neoplasia Risk

### 2.1. Epidemiological Evidence

The latest meta-analysis on psoriasis and cancer risk, including 112 cohort studies, demonstrates a significant association between these conditions, with an overall cancer prevalence of 4.78% (95% CI, 4.02–5.59%) among patients with psoriasis [7]. One study indicates that the overall cancer risk increases with the duration of psoriasis [8]. The most commonly reported site-specific cancers in patients with psoriasis were skin cancer, lymphomas, urological cancer, lung cancer, and aerodigestive tract cancers [7,9,10]. According to a prospective longitudinal cohort study, women exhibited a higher standardized incidence ratio of cancer compared to men [11].

The incidence of non-melanoma skin cancers in patients with psoriasis appears to be influenced by both disease-related factors and treatments, such as PUVA therapy and cyclosporine, while biologics targeting IL-17 and IL-23, and ustekinumab, because *IL-12/23* treatment targets show no significant association with skin cancer risk [12].

The incidence of keratinocyte cancers is closely linked to UV radiation exposure. Consequently, the increased frequency of keratinocyte cancers observed in psoriasis patients undergoing PUVA therapy can be attributed to this factor. However, a study that adjusted for confounders found that the frequency of keratinocyte cancers is slightly elevated in psoriasis patients overall, regardless of PUVA therapy [13]. The same study shows that there is no correlation between the severity of psoriasis and the risk of developing non-melanocytic cancers. An unadjusted factor remains the increased frequency of dermatological consultations among psoriasis patients, which may lead to more frequent detection of skin cancer. A study from the United States [14], focused exclusively on women, demonstrates a higher incidence of squamous cell carcinoma among patients with psoriasis while finding no association between psoriasis and the risk of developing basal cell carcinoma. Based on a Danish nationwide cohort study, the increased risk of skin cancer in psoriasis patients appears to be influenced by ethnicity, particularly in populations with fair skin, such as those of Scandinavian origin. However, the study also pointed out the lack of diversity in the population analyzed, limiting the generalizability of its findings to individuals of darker skin tones or non-Scandinavian origins. The role of ethnicity in skin cancer risk among psoriasis patients underscores the need for further research in more ethnically diverse populations to understand how genetic and environmental factors intersect in determining cancer susceptibility within this group [13].

Regarding the risk of melanoma in patients with psoriasis, there is no clear vision. According to a population-based cohort study from Taiwan [15], melanoma is associated with psoriasis, particularly in younger males. However, there are studies that do not show an association between psoriasis and the risk of developing melanoma [16,17]. Furthermore, a retrospective study from Italy involving 72,739 psoriasis patients concludes that their risk of developing melanoma is lower than in the general population [18].

Psoriasis is also noted to be associated with an elevated risk of lymphomas. Non-Hodgkin lymphomas, which account for the majority of lymphomas, are classified into B-cell and T-cell lymphomas. Cutaneous T-cell lymphoma (CTCL) is a subtype of T-cell lymphoma. According to several studies, the most significant correlation between psoriasis and lymphomas is observed with cutaneous T-cell lymphoma (CTCL) [19,20,21,22]. Given that psoriasis also involves T-cell dysfunction in the skin, a potential pathophysiological link between the two conditions has been suggested [19].

As reported by several studies, patients with psoriasis have a higher tendency to develop urological tumors, including bladder, kidney, or urinary tract tumors [10,15,16]. However, a study that adjusted for alcohol and tobacco use shows that there is no significant association between psoriasis and the risk of developing bladder cancer [8].

The risk of developing lung tumors has also been cited in patients with psoriasis [10,16,23,24,25]. However, due to the strong link between smoking and lung cancer, two studies that adjusted for smoking found no association between psoriasis and lung tumors [8,26].

In a similar manner, upper aerodigestive tract cancers appear to be associated with psoriasis [10,15,27], but after adjusting for potential etiological factors such as alcohol consumption and smoking, no significant association between psoriasis and these tumors was observed [8]. Regarding colorectal cancer, several studies have reported an association with psoriasis [9,26], a finding further supported by the most recent meta-analysis on the subject [10]. The same meta-analysis also shows a link between psoriasis and liver and pancreas tumors [10].

The malignancy risk profile in patients with psoriasis and psoriatic arthritis (PsA) exhibits distinctive epidemiological patterns, with PsA research remaining less comprehensive than that of psoriasis. While the overall prevalence and incidence of cancer in PsA patients appear similar to the general population, specific malignancies show notable variations [28]. PsA is associated with a significantly increased risk of keratinocyte cancer (RR: 1.22) [29], as well as a higher incidence of melanoma (IRR: 1.36) compared to individuals with mild or severe psoriasis [13]. Moreover, recent meta-analyses have identified a heightened risk of breast cancer in PsA patients (RR: 1.73) [7].

### 2.2. Common Biological Pathways

Tumor initiation begins when normal cells acquire genetic mutations conferring growth and survival advantages. Multiple mutational events, at least four or five, are typically necessary for malignant transformation [30].

Chronic inflammation is a critical driver of tumor initiation, providing a biological environment that supports mutagenesis and genomic instability. This process begins when inflammatory cells, such as macrophages and neutrophils, release reactive oxygen species (ROS) and reactive nitrogen intermediates (RNI). These molecules can damage DNA, leading to mutations and impairing repair mechanisms. Chronic exposure to ROS and RNI exacerbates the accumulation of genetic alterations, which, in turn, promote the transformation of normal cells into tumorigenic ones. Such inflammation-induced DNA damage is often accompanied by inactivation of key tumor suppressors, such as p53 [31,32].

In addition, chronic inflammation plays a critical role in cancer initiation and progression by shaping the tumor microenvironment to promote carcinogenesis. This microenvironment comprises a dynamic network of innate immune cells (e.g., macrophages, mast cells, neutrophils, myeloid-derived suppressor cells, natural killer cells, and dendritic cells) and adaptive immune cells (e.g., T and B lymphocytes), alongside cancer cells and surrounding stromal components, including fibroblasts, mesenchymal cells, endothelial cells, and pericytes. These infiltrating immune cells release humoral mediators, such as interleukins, proteases, and matrix metalloproteinases (MMPs), which support tumor growth, angiogenesis, and metastasis [31,33,34]. The balance within this microenvironment, dictated by the abundance and activation states of its cellular components and the expression of immune mediators, determines whether the outcome will be tumor-promoting inflammation or antitumor immunity. Tumor-associated macrophages (TAMs) are among the most prevalent immune cells in the tumor microenvironment and are pivotal in promoting angiogenesis, invasion, and metastasis by suppressing antitumor immune responses through IL-10 and PG-E2 while promoting metastasis and extracellular matrix remodeling via MMPs and angiogenic factors like VEGF [31,34]. The inflammatory tumor microenvironment is orchestrated by cytokines such as IL-6, IL-1β, TNF-α, and IL-23. These cytokines sustain tumor-associated inflammation, drive immune cell recruitment, and promote tumor growth through mechanisms that include enhancing angiogenesis, inducing chemokine production, and supporting immune evasion [31].

Psoriasis pathogenesis is characterized by a dysregulated immune response involving Th1 and Th17 cells. T-helper cells promote inflammation through the secretion of key cytokines such as tumor necrosis factor-alpha (TNF-α), interleukin-17 (IL-17), and interleukin-23 (IL-23). These cytokines are considered to play crucial roles in oncogenesis, particularly in promoting tumor growth within chronic inflammatory microenvironments [6,34,35].

TNF-α is produced at higher levels in psoriatic skin and contributes to chronic inflammation by activating NF-κB, a key transcription factor that regulates genes involved in inflammation and cell survival [36,37]. In cancers, TNF-α plays a dual role, promoting tumor cell survival and proliferation in low doses while inducing cell death in higher concentrations through NF-κB activation [38,39]. This dual nature of TNF-α in psoriasis and cancer highlights the complex role of immune dysregulation in both diseases.

The IL-17/IL-23 axis plays a significant role in linking psoriasis with cancer risk. IL-17 not only promotes keratinocyte proliferation in psoriasis but also stimulates tumor growth and metastasis in cancers by activating NF-κB and STAT3 pathways. It induces cytokines such as IL-6 and VEGF, facilitating tumor cell proliferation and vascularization. Conversely, IL-17 can enhance antitumor immunity by recruiting CD8+ T cells and activating NK cells, suggesting a context-dependent role in tumor suppression [40,41,42,43,44]. While IL-12 is recognized for its tumor-suppressive properties, IL-23 potentially contributes to tumor development [45].

IFN-γ, another cytokine involved in the pathogenesis of psoriasis with antiviral, immunomodulatory, and antitumor properties, plays a protective role by enhancing cytotoxic T-cell and natural killer (NK) cell activity, thereby strengthening immune surveillance against malignant cells [46].

Keratinocyte hyperplasia and excessive reactive oxygen species (ROS) play pivotal roles in the pathogenesis of psoriasis, with significant implications for cancer development. Psoriatic lesions are characterized by increased keratinocyte proliferation, driven by mitogenic cytokines such as IL-17A and IL-23, which activate pathways like MAPK/ERK and STAT3, leading to epidermal thickening and sustained inflammation. This persistent hyperproliferative state not only disrupts normal epidermal turnover but also creates an environment conducive to genomic instability, largely mediated by ROS. The accumulation of ROS induces oxidative stress, damaging cellular components such as DNA, proteins, and lipids, which can potentiate oncogenic transformation in keratinocytes [47].

### 2.3. Genetic and Environmental Factors

Epigenetics refers to the molecular mechanisms that influence gene activity and expression without changing the underlying DNA sequence [48]. In both cancer and psoriasis, significant epigenetic modifications are observed, including changes in DNA methylation and histone modifications, which contribute to uncontrolled cell proliferation, genomic instability, immune escape, and resistance to apoptosis, collectively promoting disease progression [49,50,51,52].

Another key player in both diseases is overexpression of the keratin 17 (KRT17), which triggers immune responses in psoriasis and serves as an oncogene in cancers like oral squamous cell carcinoma, cervical cancer, and breast cancer [53,54,55,56].

Long non-coding RNAs (lncRNAs) are critically involved in the pathogenesis of both psoriasis and neoplasia, modulating immune responses, cellular proliferation, and inflammatory pathways in these conditions. For example, lncRNA PSORS1C3 is a susceptibility gene in psoriasis, while lncRNA HOTAIR is associated with chromatin remodeling in tumors, and lncRNA HULC is significantly overexpressed in hepatocellular carcinoma [57,58].

A contributing factor to the heightened cancer risk in psoriasis patients is the shared association with common risk factors. Multiple well-known cancer risk factors, including lifestyle behaviors such as smoking, alcohol consumption, and obesity, are also frequently linked to psoriasis [59,60]. Smoking, for example, is a widely recognized risk factor for several types of cancer, including lung and bladder cancer, and has been associated with psoriasis as well [61,62]. Similarly, alcohol consumption, linked to liver, esophageal, and colorectal cancers, is also observed at a higher rate among patients with psoriasis [61]. Obesity is another common risk factor that intersects both conditions. It leads to chronic low-grade inflammation, contributing to the pathogenesis of psoriasis and being a known risk factor for cancers such as colorectal and liver cancer [61]. The overlap between these cancer-related risk factors and those associated with psoriasis may help explain the higher incidence of certain cancers observed in individuals with psoriasis.

## 3. Psoriasis Treatments and Cancer Risk

### 3.1. Topical Therapy and Oral Corticosteroids

Despite the limited number of studies investigating the malignancy risk associated with topical therapies in psoriasis, existing evidence indicates no significant association between the use of topical corticosteroids or vitamin D analogs and the development of cancer [63,64,65,66].

Tazarotene, a topical retinoid used in psoriasis treatment, functions through its active metabolite, tazarotenic acid, which binds to RAR-β and RAR-γ receptors, influencing keratinocyte proliferation and differentiation. Although no studies directly evaluate its association with cancer risk, evidence highlights its effectiveness in treating basal cell carcinoma (BCC), squamous cell carcinoma (SCC), and mycosis fungoides (MF). Clinical studies have demonstrated tazarotene’s ability to upregulate the tumor suppressor gene TIG-3, which is downregulated in conditions like psoriasis, BCC, and SCC, reducing keratinocyte proliferation and enhancing apoptosis [67].

Coal tar, one of the oldest treatments for psoriasis, possesses anti-inflammatory, antibacterial, and antiproliferative properties. Although its polycyclic aromatic hydrocarbons (PAHs) are metabolized into DNA-binding metabolites with carcinogenic potential, evidence regarding cancer risk in psoriasis patients is inconsistent. While chronic exposure in occupational and animal studies has shown an increased risk of non-melanoma skin cancer, most dermatological studies do not indicate a significant association with skin or internal cancers [68,69].

A population-based study in Denmark investigated the association between oral glucocorticoid use and the risk of developing basal cell carcinoma (BCC), squamous cell carcinoma (SCC), melanoma, and non-Hodgkin’s lymphoma (NHL). The study found that oral glucocorticoid use was associated with a modestly increased risk of BCC, particularly in individuals with longer durations of use [70]. However, the study faced several limitations, including the omission of important confounding factors, such as chronic sun exposure and variations in skin phenotype, which could have impacted the findings.

### 3.2. Phototherapy

Phototherapy is a well-established treatment for psoriasis that reduces dendritic cell activity and inhibits the activation of effector T-cells, thereby modulating the immune response and decreasing inflammation [71].

Phototherapy can increase carcinogenesis risk by inducing DNA damage, such as cyclobutane pyrimidine dimers (CPDs) and 6-4 photoproducts (6-4PPs). These lesions disrupt DNA replication and, if unrepaired, contribute to skin cancer development. CPDs contribute to tumorigenic gene mutations and immunosuppression, while 6-4PPs activate pathways promoting cell survival but enable mutation accumulation. UV radiation also impairs the p53 tumor suppressor, weakening genomic defenses and facilitating malignant transformation. These mechanisms underscore the dual role of UVR in inducing mutations and compromising cellular repair systems [72].

PUVA (oral 8-methoxypsoralen plus ultraviolet A light therapy) is associated with an increased risk of non-melanoma skin cancers (NMSC), especially squamous cell carcinoma (SCC), even in areas not typically exposed to sunlight, and this risk persists even after the cessation of therapy [10]. One study shows that patients receiving more than 350 sessions of PUVA face a significantly higher risk of developing SCC [73]. The same study indicates that PUVA therapy poses a significantly lower risk for basal cell carcinoma (BCC) compared to squamous cell carcinoma (SCC), even after high-dose exposure [73]. Research indicates also that patients who undergo more than 250 sessions of PUVA are at a higher risk of developing melanoma [74]. The PUVA Follow-up Study [75] aimed to evaluate the risk of developing non-cutaneous malignancies in patients undergoing PUVA therapy. After extensive analysis, no significant association was found between PUVA therapy and the development of non-cutaneous tumors. The results suggest that, despite the established link between PUVA and an increased risk of skin cancers, particularly squamous cell carcinoma (SCC), the treatment does not appear to elevate the risk for internal malignancies.

UVB therapy, particularly narrowband UVB (NB-UVB), is a widely used treatment for psoriasis. Studies have generally shown that NB-UVB therapy carries a lower risk of skin cancer compared to PUVA therapy [76]. Most research suggests that patients receiving NB-UVB therapy do not face a significant increase in the risk of skin cancers, including basal cell carcinoma (BCC) and squamous cell carcinoma (SCC) [77]. However, some studies have suggested that high cumulative doses of UVB, particularly in patients receiving more than 300 sessions, may lead to a slight increase in the risk of SCC, especially in combination with other treatments like PUVA [78]. In contrast, data regarding the risk of melanoma following UVB therapy remains limited, and the evidence does not strongly support a significant increase in melanoma risk [78].

### 3.3. Conventional Systemic Therapies

Methotrexate (MTX) is a conventional systemic therapy commonly used for the treatment of moderate to severe psoriasis. As an analog of folic acid, methotrexate works by blocking the enzyme dihydrofolate reductase, thereby impairing DNA replication. This mechanism decreases the proliferation of keratinocytes, leading to reduced epidermal thickening. In psoriasis management, doses typically do not exceed 25 mg per week, which has immunosuppressive effects [79].

The potential malignancy risk associated with methotrexate remains under debate. A comprehensive meta-analysis of psoriasis patients undergoing methotrexate treatment reported a malignancy incidence of 1.2% [80]. According to a cohort study from Korea, there are no significant differences in the incidence of malignancies between methotrexate-treated patients and non-psoriatic individuals [81]. The finding in this study is also consistent with rheumatologic research, which also suggests that methotrexate use does not substantially increase the overall cancer risk [82,83,84].

The association between methotrexate treatment and the risk of developing skin cancer remains inconclusive and has not been definitively established. Methotrexate has been reported to be associated with an elevated risk of basal cell carcinoma (BCC) in patients with psoriasis. The PSOLAR observational study found that individuals treated with methotrexate demonstrated a higher incidence of BCC when compared to those receiving alternative therapies or no systemic treatment [85]. This elevated risk has also been documented in patients with psoriatic arthritis and rheumatoid arthritis treated with methotrexate [86]. The use of methotrexate (MTX) in patients with psoriasis, psoriatic arthritis, and rheumatoid arthritis has been associated with an elevated risk of squamous cell carcinoma (SCC) [86]. Evidence suggests that prolonged exposure to methotrexate, particularly beyond one year, significantly increases the likelihood of developing a second non-melanoma skin cancer (NMSC), with SCC being the most prominent type [86]. Furthermore, combining methotrexate with PUVA therapy has been shown to double the risk of SCC [87], while the combination of methotrexate with cyclosporine also amplifies the risk of NMSC development [88]. Extended methotrexate use, particularly over two years, is considered an independent risk factor for SCC [73]. The association between methotrexate and melanoma is less definitive. Some studies indicate a small but noteworthy increase in melanoma risk associated with methotrexate treatment [89,90]. However, other research reported no increased melanoma risk in patients treated with methotrexate [91].

Although the connection between methotrexate and lymphoproliferative disorders in psoriasis patients is less well-established, several case reports highlight the development of Epstein–Barr virus-associated lymphomas in psoriasis individuals treated with methotrexate [92,93,94]. One report described a case of a patient with severe psoriasis who received methotrexate for 15 years, developed pulmonary lymphoma, and was positive for Epstein–Barr viral RNA [95].

The association between methotrexate dosage and cancer risk suggests a dose-dependent relationship. While high-dose methotrexate is a well-established treatment for various malignancies, evidence from observational studies in rheumatoid arthritis patients indicates that low-dose methotrexate (≤20 mg/week) may contribute to an increased incidence of certain cancers. These include cutaneous malignancies, as well as hematologic and pulmonary cancers [86,96,97].

Cyclosporin (CsA) is an immunosuppressive drug classified as a traditional systemic therapy used to manage severe psoriasis, especially resistant forms of the disease. CsA is typically administered as a short-term therapy lasting between 2 and 4 months, with treatment courses repeated at intervals [79]. Cyclosporin works by inhibiting calcineurin, a key enzyme in T-cell activation. Specifically, it binds to cyclophilin, forming a complex that disrupts T-cell function by blocking the production of interleukin-2 (IL-2), thereby reducing the immune activity responsible for psoriasis progression [98,99].

Long-term use of cyclosporin is linked to an increased risk of malignancy due to its immunosuppressive effects. A cohort study involving 1252 psoriasis patients treated with cyclosporin revealed a standardized incidence ratio (SIR) of 2.1 for malignancy compared to the general population, indicating a higher overall risk [100].

Cyclosporin is strongly associated with an increased risk of non-melanoma skin cancers (NMSC), particularly SCC. The mechanism behind cyclosporin-associated skin cancer is thought to involve T-cell dysfunction and the upregulation of transforming growth factor-beta (TGF-β), which enhances the invasive activity of cancer cells [100]. Studies have shown that patients who used cyclosporin for more than two years faced a higher risk of SCC [101]. Furthermore, in patients who had previously undergone PUVA treatment, the risk of developing SCC was three times higher when cyclosporin was subsequently introduced. PUVA-treated patients with over 200 sessions exhibited the highest risk [102]. However, one retrospective cohort study conducted in Finland found no significant association between short-term cyclosporin and SCC [103].

Cyclosporin use has also been associated with an elevated risk of lymphoproliferative malignancies, particularly in patients undergoing organ transplantation. Long-term CsA therapy in transplant patients has been linked to serious side effects, including lymphoma [104,105]. The distinction lies in the dosage of cyclosporine, which is administered at significantly higher levels in organ transplant recipients compared to patients with psoriasis [79]. In contrast, psoriasis treatment typically involves variable and intermittent dosing, complicating direct comparisons. Even so, there is one case report documenting the development of B-cell lymphoma in a psoriasis patient treated with cyclosporin, where the lymphoma occurred seven months after discontinuation of an eight-month treatment [106]. A study focusing on psoriasis patients reported that among those who developed cancer, 85% had received medium doses of cyclosporine (2–4 mg/kg) [104].

Acitretin is a synthetic retinoid commonly used to treat psoriasis due to its ability to normalize epidermal proliferation [79]. When it comes to cancer risk, the data regarding the potential of acitretin to cause skin cancers is limited. Some studies suggest that acitretin may actually reduce the risk of squamous cell carcinoma (SCC), especially when used in combination with PUVA therapy. This combination seems to decrease the toxicity of PUVA, which is known to increase the risk of SCC [107]. Acitretin has also been investigated for its prophylactic use in preventing skin cancers in post-transplant patients, further highlighting its potential to reduce skin cancer risk [108,109]. While studies assessing the effect of acitretin on internal organ malignancies are sparse, current evidence suggests that acitretin may not increase overall cancer risk, as it lacks immunosuppressive effects [81].

Fumaric acid esters have been used as a systemic therapy for psoriasis since 1994, particularly in Germany and northern European countries. Their effectiveness in treating psoriasis vulgaris has been recognized since 1959, and they are commonly prescribed for severe forms of the disease [79]. Current evidence suggests that oral fumaric acid esters do not significantly increase the overall risk of malignancy in patients with psoriasis [59]. Several studies conducted in Germany have found no correlation between the use of fumaric acid esters and the development of non-melanoma skin cancer (NMSC) [110,111]. However, one study has raised concerns about a potential association between fumaric acid ester treatment and the risk of melanoma [112].

### 3.4. Biologic Agents

Biologic therapy has transformed the management of psoriasis, offering targeted and effective treatment options. Currently, four categories of biologics are available: tumor necrosis factor inhibitors such as adalimumab, etanercept, infliximab and certolizumab; interleukin-12/23 antagonists like ustekinumab; IL-17A inhibitors including ixekizumab and secukinumab, as well as IL-17 receptor antagonists like brodalumab; and anti-IL-23 agents such as risankizumab, tildrakizumab, and guselkumab.

The available evidence on cancer risk in patients with psoriasis undergoing biologic monotherapy compared to combination therapy with biologics and non-biologics is scarce. However, a retrospective cohort study utilizing data from Taiwan’s National Health Insurance Research Database (2010–2020) assessed cancer risk in psoriasis and psoriatic arthritis patients treated with monotherapy or combination therapy involving biologics and non-biologics. Among biologic-treated patients, 78.4% received combination therapy, with 30% receiving tumor necrosis factor inhibitors. The overall risk of hematologic malignancies did not differ significantly between patients treated with non-biologics and those receiving combination therapy of biologics and non-biologics [113].

The introduction of biologic therapies has raised concerns about an increased risk of malignancy. Limited data, particularly regarding IL-17/IL-23 inhibitors, in real-world contexts complicates their application. However, evidence is emerging to support their potential [114]. However, several studies have not demonstrated a heightened risk of cancer in psoriasis patients treated with biologics. A meta-analysis from multiple countries reported no elevated cancer risk with long-term biologic exposure [115]. Despite these reassuring findings, the potential risks remain a topic of ongoing investigation.

TNF-alpha exhibits both pro- and antitumor effects by mediating apoptosis and promoting carcinogenesis within the tumor microenvironment; however, TNF-alpha inhibitors, by suppressing its dual role, may impair immune surveillance, enhance tumor cell survival through NF-kB activation and increase the risk of malignancies [116].

Studies specifically examining the malignancy risk associated with TNF inhibitors (TNFi) have shown mixed results. While most research does not demonstrate a significant increase in overall cancer risk, some studies have observed an increased incidence of NMSC, particularly squamous cell carcinoma (SCC), in patients treated with TNF inhibitors compared to the general population [117,118]. This elevated risk of SCC is thought to be influenced by prior treatments like cyclosporine or PUVA [119]. Notably, some case reports highlight the rapid onset of SCC in patients receiving TNFi, such as a case of SCC developing on the penis of a patient taking etanercept [120,121]. The association between TNFi treatment and basal cell carcinoma (BCC) is less clear, but some studies have reported an increased risk. For example, the PSOLAR observational study found a higher incidence of BCC in psoriasis patients treated with TNFi, although the risk of SCC was not similarly elevated [122]. The risk of developing melanoma in TNFi-treated patients remains uncertain, with mixed findings from various studies. While some large-scale studies, such as those conducted across European biologic registries, have found no significant increase in melanoma risk among TNFi users [110,123,124], case reports and small cohort studies suggest a possible association, particularly with adalimumab [125,126,127].

The use of TNF inhibitors has also been linked to an increased risk of lymphoproliferative disorders, such as non-Hodgkin and Hodgkin lymphomas [128]. Some case reports have described the development of lymphomas, including cutaneous T-cell lymphomas (CTCL), in patients treated with TNFi [129]. Furthermore, a case of gastric mucosa-associated lymphoid tissue lymphoma was reported in a psoriasis patient treated with infliximab [130]. Additionally, there has been a case report documenting the development of myelodysplasia with acute myeloid leukemia in a psoriasis patient following treatment with TNFi [131].

Etanercept has been explored in cachectic cancer patients for its potential to alleviate cancer anorexia and weight loss syndrome by blocking TNF-α’s interaction with cell surface receptors. Although preclinical data suggest therapeutic value, clinical trials have demonstrated limited efficacy, showing no significant improvement in appetite, weight gain, or survival outcomes. Additionally, concerns persist about its potential adverse effects and the immunosuppressive risks associated with TNF-α inhibition, which could influence its overall risk-benefit profile in cancer patients [132].

Ustekinumab is an interleukin-12/23 antagonist used in the treatment of severe psoriasis. IL-12 promotes antitumor activity, largely through the activation of IFN-γ, while IL-23 has tumor-promoting effects, facilitating the epithelial–mesenchymal transition and increasing tumor cell migration. These contrasting roles highlight that the use of anti-IL-12/23 therapies, such as those targeting the shared p40 subunit, could alter the tumor microenvironment, potentially increasing the risk of malignancy by reducing IL-12-driven antitumor effects [133]. A Korean population-based study showed a reduced overall cancer risk in psoriasis patients treated with ustekinumab [134]. Furthermore, long-term safety studies have not demonstrated an increased risk of non-melanoma skin cancers or melanoma compared to the general population [135,136]. Although some cases of cutaneous T-cell lymphoma have been associated with ustekinumab use, these instances are rare, and the overall cancer risk remains similar to that seen in the placebo-treated group [129].

Interleukin-17 exhibits a dual role in cancer, acting as both a tumor-promoting and tumor-suppressing factor depending on the specific context. IL-17 promotes tumorigenesis through its involvement in chronic inflammation, angiogenesis, and immune evasion, enhancing tumor cell survival and metastasis. It activates pro-inflammatory pathways, such as NF-κB and STAT3, leading to the production of cytokines like IL-6 and VEGF that support tumor growth and vascularization. IL-17 also recruits myeloid-derived suppressor cells and inhibits cytotoxic T-cell infiltration, fostering an immunosuppressive tumor microenvironment. However, IL-17 can also facilitate antitumor immunity by attracting CD8+ T cells and enhancing natural killer cell activity under specific conditions. The dual nature of IL-17 raises concerns about the potential oncogenic risks of anti-IL-17 therapies used in psoriasis treatment [42,43,44].

The overall cancer risk associated with IL-17 inhibitors appears to be low. Studies evaluating patients treated with IL-17 inhibitors suggest that this class of drugs does not significantly increase the risk of malignancies [137]. In fact, a large population-based study demonstrated that IL-17 inhibitors were associated with a reduced risk of several cancers, including lymphoproliferative disorders, solid organ cancers, and basal cell carcinoma (BCC), when compared to tumor necrosis factor inhibitors (TNFi), also suggesting a protective role of IL-17 inhibition in reducing tumor progression [137].

The evidence on the risk of squamous cell carcinoma in patients treated with IL-17 inhibitors is limited. IL-17 inhibitors appear to reduce the risk of developing basal cell carcinoma. A recent population-based study found that patients treated with IL-17 inhibitors had a decreased risk of BCC compared to those treated with TNFi [137]. The risk of melanoma in patients treated with IL-17 inhibitors remains a subject of investigation. While the study mentioned above reported a reduced risk of melanoma in IL-17 inhibitor-treated patients compared to TNFi-treated patients, the overall data on melanoma risk are still limited and warrant further long-term studies.

A large international study indicated a decreased risk of non-Hodgkin lymphoma in patients treated with IL-17 inhibitors [137]. However, there have been a few case reports of cutaneous T-cell lymphoma (CTCL) associated with IL-17 inhibitors, specifically secukinumab and ixekizumab [129].

IL-23, produced by activated dendritic cells and macrophages, promotes T-helper 17 (Th17) differentiation and subsequent IL-17 production, which can support tumor-promoting inflammation. Additionally, IL-23 suppresses natural killer cell-mediated tumor surveillance, exerting a pro-tumor effect independent of IL-17A. Its downstream signaling, involving STAT3 and STAT4 activation, determines whether pro- or antitumor effects dominate, depending on factors such as genetic background, tumor type, and environmental triggers [138,139].

There are limited studies on the cancer risk in psoriasis patients treated with IL-23 inhibitors, but current evidence suggests that IL-23 inhibitors may offer a favorable safety profile regarding cancer development [137]. For example, a retrospective study by Gargiulo et al. underscores the potential utility of IL-17 and IL-23 inhibitors in managing moderate-to-severe psoriasis in oncologic patients, demonstrating significant clinical efficacy without evidence of malignancy reactivation or progression during a median follow-up of over two years. Despite the occurrence of new neoplasms in a subset of patients, no causal link to IL-17 and IL-23 inhibitors was established, reflecting their promising safety profile [114]. In a systematic review of cutaneous T-cell lymphoma (CTCL) cases associated with biologic therapies, no cases of CTCL were reported in patients treated with IL-23 inhibitors for plaque psoriasis [129].

The progression of neoplasia in psoriasis patients treated with biologic therapies remains a critical concern due to the immunomodulatory nature of these agents. Evidence suggests varied outcomes depending on the class of biologic therapy and the stage of malignancy.

According to a retrospective observational study [140], anti-TNFα agents showed minimal association with cancer progression, with only one case reported in the target population. In another study [141], anti-IL-12/23 therapy has shown a generally favorable safety profile, with only isolated reports of cancer progression, such as a case of esophageal cancer progression during treatment. One study suggests that anti-IL-17 therapies have a dichotomous pattern: no cancer recurrence was observed in early-stage or remission cases, while progression was noted in advanced malignancies [142]. A retrospective observational study indicated that anti-IL-23 therapies were linked to an elevated rate of cancer progression in patients receiving concurrent oncologic treatment, with progression reported in four out of seven cases, two of which culminated in patient mortality [140].

### 3.5. New Therapies

Apremilast is a small-molecule inhibitor targeting phosphodiesterase 4 (PDE4), an enzyme that degrades cyclic adenosine monophosphate (cAMP). By inhibiting PDE4, apremilast increases intracellular cAMP levels, which helps regulate the balance between pro-inflammatory and anti-inflammatory mediators. This action reduces the production of inflammatory cytokines such as TNF-α, IL-17, and IL-23, which are central to psoriasis pathogenesis [143]. Apremilast appears to have a favorable safety profile for psoriasis patients, even those with a history of malignancy. In a case series and literature review covering 841 patients with serious comorbidities, including cancer, apremilast was not associated with a significant increase in cancer progression or recurrence. This suggests that apremilast may be a low-risk option for psoriasis patients concerned about cancer risks [144].

Deucravacitinib acts as a selective inhibitor of tyrosine kinase 2 (TYK2) by binding to its regulatory (pseudokinase) domain, thereby preventing TYK2 activation while sparing the activity of other Janus kinase (JAK) family members. TYK2 mediates signaling for cytokines such as IL-23 and type I interferons, both critical in psoriasis. By selectively inhibiting TYK2, deucravacitinib reduces IL-23-mediated Th17 cell activation and the downstream inflammatory cascade, leading to decreased keratinocyte proliferation and inflammation [145]. Deucravacitinib is approved for the treatment of moderate and severe psoriasis [146]. It showed a favorable safety profile with low malignancy rates across clinical trials. Over the 52-week study period, the incidence of malignancies among patients treated with deucravacitinib was comparable to those treated with placebo or apremilast, suggesting no increased cancer risk associated with its use [147,148].

Tofacitinib is an oral medication classified as a Janus kinase (JAK) inhibitor, approved by the FDA for the treatment of psoriatic arthritis (PsA) [149]. Although it is not currently approved for the treatment of psoriasis, clinical studies have demonstrated its efficacy in managing this condition [150,151].

Tofacitinib functions by reversibly inhibiting the ATP-binding site of JAK proteins, specifically targeting JAK1, JAK3, and, to a lesser extent, JAK2. This inhibition prevents the activation of STAT proteins, which are essential for the transcription of pro-inflammatory genes implicated in psoriasis. By disrupting the JAK-STAT signaling pathway, tofacitinib reduces the activity of cytokines that promote inflammation and keratinocyte proliferation, such as interleukins and interferons. This results in decreased T-cell activation and a reduction in psoriatic symptoms [152].

Clinical trials and studies have reported incidences of both non-melanoma skin cancers (NMSCs) and other malignancies in patients receiving tofacitinib for psoriatic arthritis and psoriasis [152,153,154]. In clinical studies involving patients with PsA, five cases of malignancies (excluding NMSC) were reported among those treated with tofacitinib, resulting in an incidence rate (IR) of 0.6 per 100 patient-years (95% CI: 0.2–1.5) [153]. The reported malignancies included bladder transitional cell carcinoma, renal cell carcinoma, metastatic pancreatic cell carcinoma (which resulted in death), squamous cell carcinoma of the vulva, and invasive ductal breast carcinoma. Four cases of NMSC were also reported (IR: 0.5; 95% CI: 0.1–1.3), comprising two basal cell carcinomas and two squamous cell carcinomas. In studies focusing on psoriasis treatment, malignancies reported during tofacitinib therapy included melanoma, esophageal carcinoma, prostate cancer, colon cancer, pancreatic cancer, and breast cancer. The rates of NMSC were relatively low, with cases of basal cell carcinoma and squamous cell carcinoma reported infrequently [151].

Upadacitinib is an oral selective Janus kinase (JAK) inhibitor with high specificity for JAK1 and moderate activity on JAK2, approved for the treatment of psoriatic arthritis (PsA) [155,156,157].

Although it is not approved for psoriasis, clinical trials, including SELECT-PsA 1 and SELECT-PsA 2, have demonstrated its efficacy in improving psoriatic symptoms in patients with PsA, indicating its potential utility in psoriasis management [158,159].

The potential malignancy risk associated with upadacitinib has been evaluated in clinical trials, primarily for PsA. In the SELECT-PsA 2 study, three cases of malignancies were identified in each upadacitinib dose group: 15 mg (including prostate cancer, basal cell carcinoma, and rectal cancer) and 30 mg (comprising ovarian and endometrial cancer, rectal adenocarcinoma, and basal cell carcinoma). These events occurred within six months of initiating treatment, suggesting a possible predisposition rather than a direct causal link to upadacitinib [159].

Extended analyses, including SELECT-PsA 1, confirmed that malignancy rates, including NMSC, remained low and comparable to other biologics, with no observable patterns suggesting a time-dependent relationship. Across studies, the rates of malignancy with upadacitinib were consistent with those observed for other DMARDs and biologics used in PsA and psoriasis [151]. However, long-term data are necessary to fully elucidate the malignancy risk profile associated with upadacitinib treatment.

## 4. Psoriasis Management in Oncologic Patients

### 4.1. Management Strategies for Psoriasis in Patients with Malignancy

In patients with psoriasis and a history of cancer, treatment selection must be approached cautiously. Biologic therapies, particularly TNF-alpha inhibitors, are often avoided due to concerns about their impact on cancer progression or recurrence. However, recent studies suggest that IL-17 and IL-23 inhibitors might offer a safer option. The EuroGuiDerm guidelines recommend considering topical therapies, NB-UVB (except for patients at risk of skin neoplasia), or non-immunosuppressive therapies such as acitretin or low-dose methotrexate for psoriasis management in patients with a history of cancer [160].

According to recent studies, apremilast offers distinct advantages as a treatment option. Unlike other systemic therapies and biologics, apremilast is not linked to significant immunosuppressive effects, making it a safer choice for individuals with cancer concerns. It is not contraindicated for patients with active or past malignancies, which allows for broader use in those who may be at risk of cancer progression. Additionally, apremilast does not require routine laboratory monitoring, adding a level of convenience and reducing the treatment burden. This profile is especially beneficial in oncologic patients with psoriasis, providing effective management of skin symptoms without compromising their cancer treatment outcomes or elevating malignancy-related risks [144].

The selective inhibition of TYK2 by deucravacitinib avoids broader immunosuppression linked to other JAK inhibitors, making it a promising option for individuals with cancer concerns. Its targeted action potentially reduces the risk of cancer progression or recurrence while still providing significant improvements in skin symptoms and quality of life when compared to both placebo and apremilast [147,148].

### 4.2. Optimal Timing of Psoriasis Treatment Relative to Cancer Diagnosis

The timing of psoriasis treatment relative to cancer diagnosis is crucial. The determination of a definitive safe timing for psoriasis treatment after cancer diagnosis remains unclear. This uncertainty arises from the variability in the types of neoplasms, their prognoses, and the different stages at which patients received treatment, ranging from 1 to 10 years post-cancer diagnosis. For patients with a history of malignancy, a waiting period of at least five years is generally recommended before initiating biologic therapies [141]. However, the use of IL-17 and IL-23 inhibitors is increasingly considered due to their favorable safety profiles in the context of malignancy.

### 4.3. Concurrent Administration of Anti-Psoriatic and Anti-Cancer Therapies

Chemotherapy and targeted anti-cancer treatments have significant immunomodulatory effects, which can influence psoriasis.

Chemotherapeutic agents, such as topoisomerase inhibitors and antimicrotubule agents, stimulate immune responses by enhancing antigen presentation and promoting immunogenic cell death. This immune modulation can lead to varying outcomes in psoriasis patients, from improvement to exacerbation during cancer treatment [161].

Immune checkpoint inhibitors, including anti-PD-1 and anti-PD-L1 therapies, are widely used for various solid tumors. However, these therapies can trigger psoriasis flares. Effective management of such flares is critical to allow continued cancer treatment. Medications like apremilast, which downregulates pro-inflammatory cytokines, and acitretin are commonly used to control psoriasis flares during immunotherapy [162,163].

The management of psoriasis in cancer patients requires a multidisciplinary approach, considering the cancer prognosis. For patients with a favorable cancer outlook, systemic psoriasis treatments are expected to have similar outcomes to those in non-cancer patients. For those with a poorer prognosis, the focus shifts to improving quality of life, with the potential benefits of psoriasis treatment outweighing the risks of cancer progression [164].

## 5. Conclusions

This review highlights the intricate relationship between psoriasis and malignancy, emphasizing the increased risk of site-specific cancers among psoriasis patients. Shared biological pathways, including chronic inflammation driven by TNF-α, IL-17, and IL-23, as well as overlapping genetic and environmental risk factors, underscore the connection between these diseases. Psoriasis treatments, particularly systemic therapies like methotrexate, cyclosporin, and TNF inhibitors, carry varying levels of cancer risk, predominantly for non-melanoma skin cancers and lymphoproliferative disorders. In oncologic patients with psoriasis, careful management is required to balance effective psoriasis control with minimizing cancer progression risk. The emerging use of IL-17 and IL-23 inhibitors and new therapies, such as PDE4 inhibitors and TYK2 inhibitors, offers promising treatment alternatives with favorable safety profiles. Ultimately, a tailored, multidisciplinary approach is essential for optimizing outcomes in psoriasis patients with a history of malignancy, ensuring that both conditions are managed effectively without compromising patients’ quality of life.
